# Synthesis of nanocellulose aerogels and Cu-BTC/nanocellulose aerogel composites for adsorption of organic dyes and heavy metal ions

**DOI:** 10.1038/s41598-021-97861-9

**Published:** 2021-09-17

**Authors:** Nuhaa Shaheed, Shahrzad Javanshir, Maryam Esmkhani, Mohammad G. Dekamin, Mohammad Reza Naimi-Jamal

**Affiliations:** 1 Pharmaceutical and Heterocyclic Compounds Research Laboratory, Department of Chemistry, Iran University of Science and Technology, Tehran, Iran; 2 Research Laboratory of Green Organic Synthesis and Polymers, Department of Chemistry, Iran University of Science and Technology, 16846 Tehran, Iran

**Keywords:** Pollution remediation, Materials science

## Abstract

MOFs compounds with open metal sites, particularly Cu-BTC, have great potential for adsorption and catalysis applications. However, the powdery morphology limits their applications. One of the almost new ways to overcome this problem is to trap them in a standing and flexible aerogel matrix to form a hierarchical porous composite. In this work, Cu-BTC/CNC (crystalline nanocellulose) and Cu-BTC/NFC (nanofibrillated cellulose) aerogel composites were synthesized using a direct mixing method by the addition of Cu-BTC powder to the liquid precursor solution followed by gelation and freeze-drying. Also, pure nanocellulose aerogels (CNC and NFC aerogels) have been synthesized from cellulose isolated from peanut shells. Scanning electron microscopy (SEM), Fourier transform infrared (FT-IR) spectra, and X-ray diffraction (XRD) were utilized to evaluate the structure and morphology of the prepared materials. The adsorption ability of pure CNC aerogel and Cu-BTC/NFC aerogel composite for organic dye (Congo Red) and heavy metal ion (Mn^7+^) was studied and determined by the UV–Vis spectrophotometry and inductively-coupled plasma optical emission spectrometry (ICP-OES), respectively. It was concluded that Cu-BTC/NFC aerogel composite shows excellent adsorption capacity for Congo Red. The adsorption process of this composite is better described by the pseudo-second-order kinetic model and Langmuir isotherm, with a maximum monolayer adsorption capacity of 39 mg/g for Congo Red. Nevertheless, CNC aerogel shows no adsorption for Congo Red. Both CNC aerogel and Cu-BTC/NFC aerogel composite act as a monolith standing solid reducer, which means they could remove permanganate ions from water by reducing it into manganese dioxide without releasing any secondary product in the solution.

## Introduction

Metal–organic frameworks (MOFs) are porous crystalline polymer networks of metal nodes (metal ions or clusters) connected to multidentate organic linkers, which are themselves linked by strong covalent bonds forming one-, two-, or three-dimensional networks^1–3^. MOFs are also known as porous coordination polymers (PCPs) or porous coordination networks (PCNs)^4–6^. In addition to combining the beneficial properties of organic and inorganic ingredients, they show unique properties that exceed expectations of a simple mixture of these parts^5,7^. Due to their specific characteristics including their record-breaking surface areas (more than 7000 m^2^/g)^8^, ultrahigh porosities^9^, low density^10^, high thermal stability^11^, and tunable pore structure^12^, MOFs have received specific attention for many applications such as gas separation and storage^13^, catalysis^14^, adsorption^15,16^, energy storage^17^, drug delivery^18–20^, chemical sensing^21,22^ and so on^23^.

Among the various types of MOFs, copper benzene tricarboxylate Cu-BTC or Cu_2_(BTC)_3_ (also called HKUST-1 or MOF-199)^24,25^ is one of the distinguished structure together with the IRMOF series. Cu-BTC was first reported in 1999 by Chui et al.^26^ and has attracted considerable attention both theoretically and experimentally^27^. Due to its open metal sites and large pore windows^28^, Cu-BTC has particular potential for adsorption and catalysis^29,30^. However, the powdered morphology of Cu-BTC, like other MOF compounds, restricts its applications^31^. It is difficult to handle such powder due to dust formation. Also, these powders cause mass transfer limitations and high-pressure drops. Different approaches and structuring strategies have been proposed to solve the above problem. Among these can be mentioned: the combination of magnetic microspheres loaded with MOF^32,33^, the synthesis of metal–organic aerogels, and the creation of a suitable substrate (such as metal oxide layers or polymeric materials)^34^ to obtain MOF compounds in a standing position.

The 1990s were a turning point in the development of controlled porous materials with successive discoveries of micro-meso and meso-macrostructure by the soft path. The porous objects with a hierarchical structure resulted from the combination of meso and macrostructure made it possible to couple properties linked to different dimensions. This effortless and green strategy permits the creation of multimodal porous materials by combining micro-mesoporous MOF and meso-macro-porous aerogels leading to MOF/aerogel composites (MOFACs)^35^.

Aerogels are ultra-low-density^36^, dry, and very porous monolithic materials^37^ taken from a gel composed of nanoparticles or polymers that chemically or physically cross-linked to a three-dimensional network^38^. Typically there are two stages for the synthesis of these materials, which are, first, the formation of wet-gels through the process of sol-gel^39^, and then drying the obtained gels using a method that keeps the internal porous morphology as possible^38,40^. The aerogel, first synthesized in the late 1930s by Kistler^41^, can be inorganic^42^, organic^43^, and hybrid^44^ aerogels according to their composition^45^. Silica aerogel is the most common inorganic aerogels, whereas resorcinol–formaldehyde aerogels are the most common organic aerogels prepared via the sol–gel process^46^. However, due to the fragility and brittleness of inorganic aerogels, organic aerogels (from natural or synthetic polymers) that are more flexible and less brittle are used if mechanical strength is required^47^. Renewable biopolymers such as cellulose are an economical and environmentally friendly alternative to synthetic polymers from reduced oil resources^36^. Although wood is the most important source of cellulose, due to competition from various sectors such as construction products, pulp, and paper industry, furniture industry, etc., the required amount of wood has been challenged for all users. As a result, agricultural by-products such as peanut shells can be a valuable source of natural cellulose fiber due to their renewable nature^24^.

Cellulose is a promising polymer for the preparation of aerogel compounds due to its unique properties such as inexpensive^48^, renewable^49^, degradable^50^, abundant^51^, non-toxic^52^, and environmentally friendly^53^. In particular, nanocellulose materials show a specific promise for use in aerogel materials, since by reducing the size of cellulose fibers, completely uniform materials with advanced mechanical properties can be obtained^15,19^. Nanocellulose aerogels have a high surface area and low density compared to regenerated cellulose aerogels^54^.

Nanocellulose aerogels and MOF are relatively new classes of nanostructured materials, and there are only two examples of literature that combine these two materials. For example, Zhu et al. synthesized MOF/CNC aerogel composite for various MOFs, including [UIO-66, ZIF-8, and MIL-100 (Fe)] by direct mixing method^23^. In another study, Zhu et al. synthesized MOF/NFC aerogel composite by the in-situ method^31^.

In this work, we report a facile and novel method to combine Cu-BTC and structural nanocellulose into flexible and porous aerogels. Two aerogel composites containing 33 wt% of distributed Cu-BTC were developed. These are Cu-BTC/NFC and Cu-BTC/CNC aerogels, which were synthesized through a straightforward sol–gel process followed by freeze-drying. Also, we synthesized the two pure nanocellulose aerogels, i.e., physically cross-linked NFC aerogel and chemically cross-linked CNC aerogel from cellulose isolated from peanut shells using *N,-N'*-methylenebisacrylamide (MBA) as a linker. The ability of these aerogels in the adsorption of both organic dyes and heavy metal ions was investigated.

## Results and discussion

### Preparation and structural characterizations of pure nanocellulose aerogels and Cu-BTC/Nanocellulose aerogel composites

Pure microcrystalline cellulose MCC was extracted from peanut shells using a combination of chemical treatments. The resulting microcrystalline cellulose fibers include amorphous and crystalline regions^47^.

When MCC was treated with concentrated sulfuric acid, the acid diffused preferentially into the amorphous regions. The available glycosidic bonds were hydrolyzed, and as a result, individual crystallites transversely were released. The dispersion of the produced CNC in water was promoted by the charged sulfate esters groups onto the surface, which were generated by the reaction between hydroxyl groups of MCC and sulfuric acid^28^. Although the resulting CNC could be turned into a gel by many physical methods, the next physical cross-linked aerogel would not have enough strength. Gelation of the CNC suspension was occurred by using MBA (*N, N'*-methylene bisacrylamide) as a linker without modifying CNCs. In this case, CNC particles play the role of Michael donors and MBA as the role of Michael acceptors. So, MBA attaches CNC particles forming a gel. This gel was aged to increase its mechanical strength by increasing the number of bonds formed between CNC and MBA particles. After freeze-drying, the obtained CNC aerogel could approach the high porosity and surface area of silica aerogels but is much less fragile.

On the other hand, the longer and more flexible nanofibrillated cellulose (NFC) was produced by the treatment of MCC suspension through a high-intensity ultrasonic method (HIUS purely mechanical process). Ultrasonic waves break the hydrogen bonds between MCC chains and lead to produce long neutral fibrillated cellulose chains with nanoscale widths and abundant hydroxyl groups on the surface of them. In this case, the resulting NFC has a gel-like state. Self-assembly without manual intervention spontaneously organized small building blocks into the nanostructure through interactions such as entanglements, intense Van der Waals forces, and hydrogen bonds. After solvent-exchange and freeze-drying, the NFC aerogel was obtained.

**Scheme 1:**
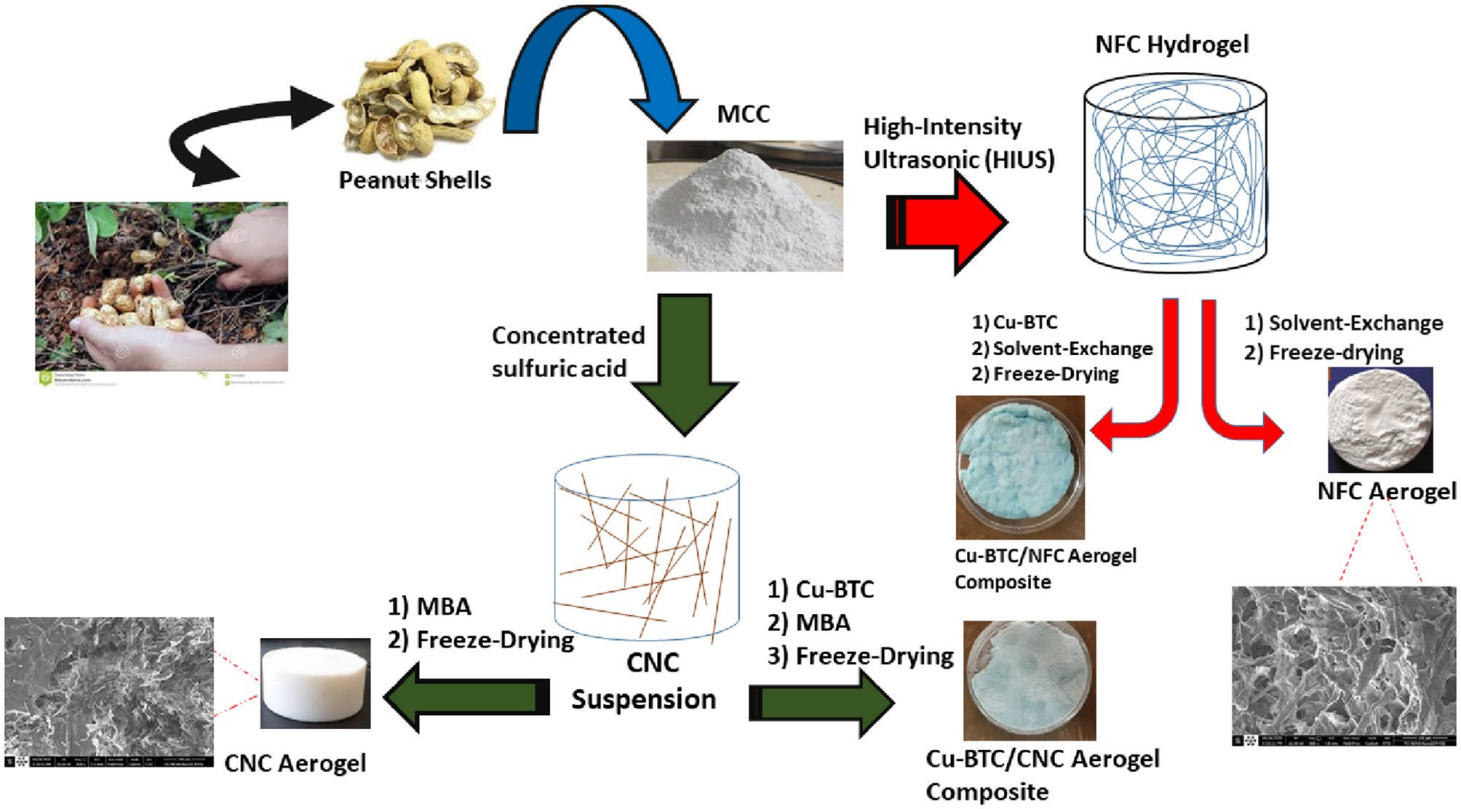
Graphical representation of the fabrication of Cu-BTC/nanocellulose aerogel composites and pure nanocellulose aerogels from peanut shells.

For fabrication of Cu-BTC/Nanocellulose aerogel composites, although a more uniform distribution of Cu-BTC crystals inside the composite can be achieved with the in-situ technique when compared to the direct mixing method, Cu-BTC growth is not easily controlled. The concentration of precursors and reaction environments, such as temperature, are generally adjusted to control Cu-BTC growth inside the pores. Moreover, the polymer network has limited porosity for mass diffusion, and the sufficient exchange of metal ions for crystal growth takes a long time (> 2 days). Consequently, the direct mixing method may be a better choice^33^, in which, Cu-BTC powder was added to aerogel precursors. According to the type of precursors, the resulting solution was changed into the gel with and without a linker. In this case, cellulosic gel clusters grew upon Cu-BTC crystals, and as a result, Cu-BTC crystals were entrapped on and inside the nanocellulose aerogel chunks. The resulting bonds between Cu-BTC crystals and nanocellulose aerogels are physical entanglement and hydrogen bonds, as well as van der Waals forces. It is important to note that all gel materials were aged before freeze-drying. During the aging period, the intermolecular bonds increase, and as a result, the mechanical strength of the subsequent aerogel material increases. A graphical representation of the fabrication of the composites and pure nanocellulose aerogels from peanut shells is described in Scheme 1. The prepared materials were characterized and compared as follows. The resulting nanocellulose aerogels are white, while Cu-BTC/Nanocellulose aerogel composites are sky blue, which is compatible with pure Cu-BTC color.

**Figure 1:**
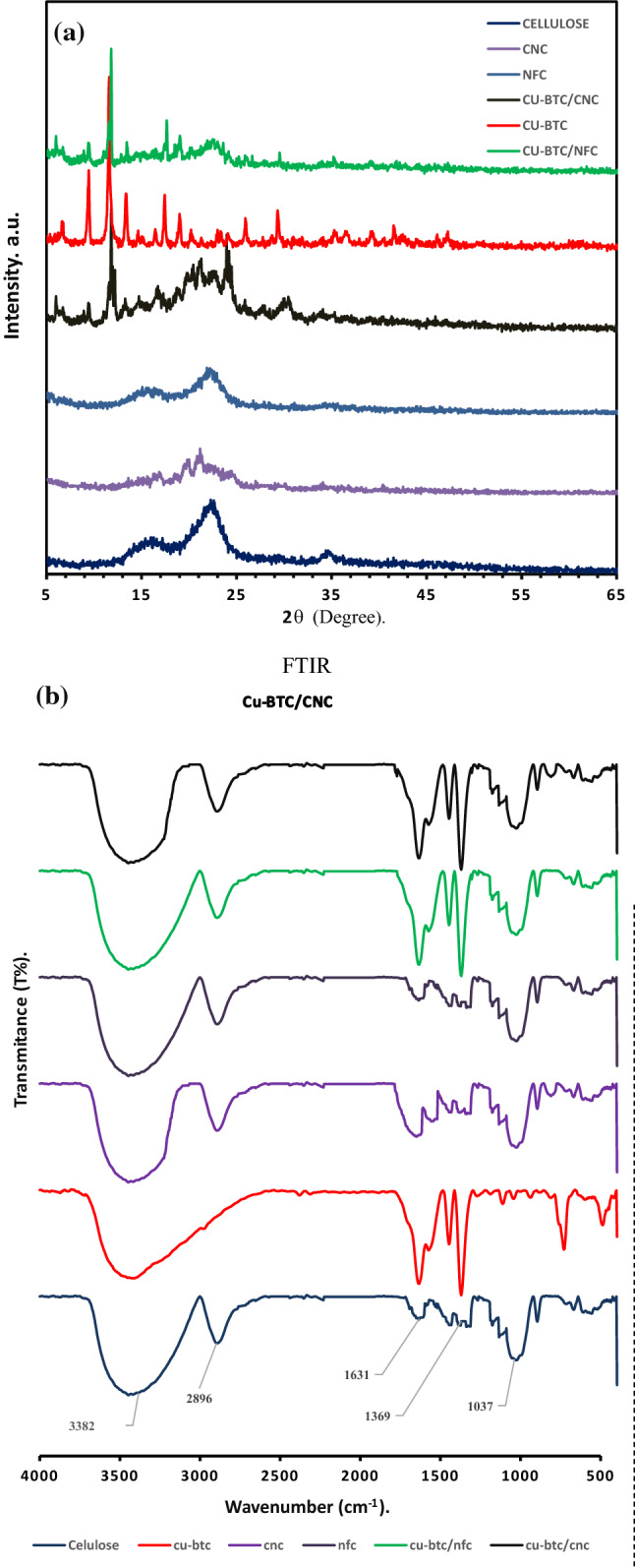
Characterization of prepared materials: (**a**) PXRD patterns, (**b**) FTIR spectra.

Figure 1a shows the powder X-ray diffraction (PXRD) of the prepared materials. As shown, the X-ray diffraction diagram of the MCC exhibited a strong peak at 2θ = 22.6°, and two overlapped weaker diffraction peaks at 2θ = 15.1° and 16.6°, besides a small diffraction peak at 34°, which are typical of cellulose I crystalline structure. The only difference between the spectrum of CNC aerogel and that of MCC is, the peak at 2θ = 22.6 splits into two weaker diffraction peaks at 2θ = 20.0 and 21.9, indicates that the crystal structure of native cellulose (cellulose I) partially converted to cellulose II during hydrolysis of sulfuric acid. The peaks of NFC aerogel spectra became less intense, indicating the crystallinity of NFC aerogel has decreased, but the crystalline structure of native cellulose did not change. In Cu-BTC/nanocellulose aerogel composites, in addition to CNC and NFC diffraction peaks, Cu-BTC diffraction peaks are present. As a result, Cu-BTC/CNC aerogel composite was prepared successfully by direct mixing without Cu-BTC crystallization disturbance.

Figure 1b illustrates the FTIR spectra of the prepared materials. In the MCC spectra, the specific peaks presented at 3354, 2893, 1639, 1434, 1168, 1050, and 893 cm^−1^ are all the generic bands of cellulose molecules. The characteristic peaks of MCC were in good agreement with those reported elsewhere^55^, indicating that microcrystalline cellulose has no impurity. No significant differences were observed in the spectrum NFC aerogel compared with that of MCC, indicating that the cellulose molecular structure did not change after HIUS treatment and freeze-drying.

Compared to MCC, the spectrum of CNC aerogel shows three new peaks at 810 cm^−1^ attributed to symmetrical C–O–S vibration associated with the C–O–SO_3_ group, and at 1544 cm^−1^ attributed to the bending vibration of N–H, as well as a characteristic peak at 1658 cm^−1^, which could be attributed to the stretching vibration of C=O, overlapping with that adsorbing water. These results confirmed that the CNC particles chemically cross-linked with each other by the MBA linker.

The FTIR spectra of Cu-BTC shows the asymmetric stretching of the carboxylate group of H_3_BTC ligand appearing at 1631 cm^−1^, and the symmetric stretching vibrations of it arise at 1573 cm^−1^. Besides, several bonds located at 600–1300 cm^−1^ attributed to the out-of-plane vibrations of BTC^3−^ anions. The band at around 3687 cm^−1^ and 2761 cm^−1^ resulted from surface-adsorbed water. The results are consistent with those reported in the literature^30,31^. In the FTIR spectra of Cu-BTC/nanocellulose aerogel composites compared to that of pure nanocellulose aerogels, the characteristic peaks of Cu-BTC are appeared, indicating that Cu-BTC particles had successfully entrapped into the aerogel networks.

**Figure 2:**
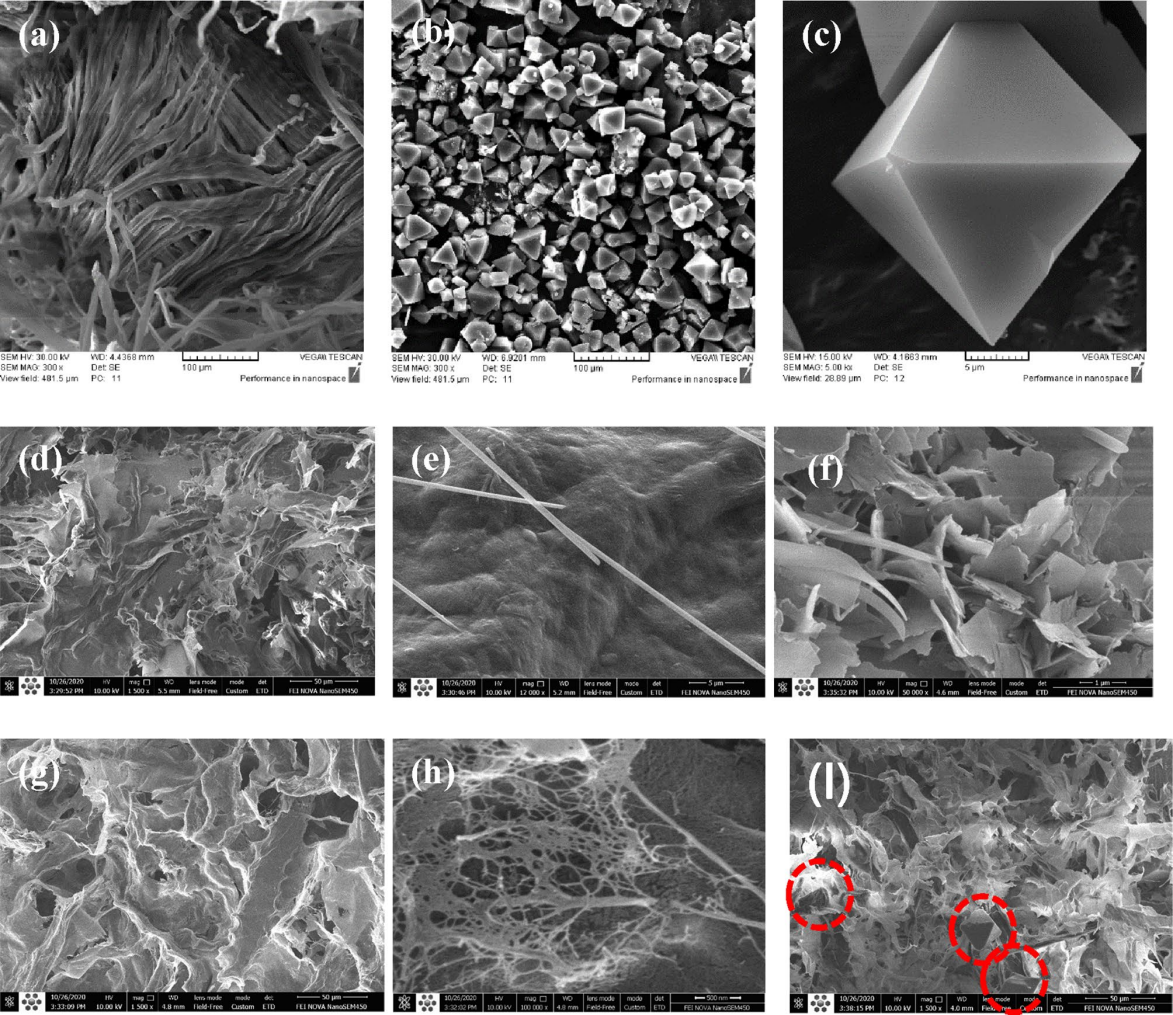
SEM images of: (**a**) MCC, (**b**,**c**) Cu-BTC at different magnification (**a** 100 μm, **b** 5 μm), (**d**,**e**) CNC aerogel at different magnification (**d** 100 μm, **e** 5 μm), (**f**) Cu-BTC/CNC aerogel composite, (**g**,**h**) NFC aerogel at different magnification (**g** 100 μm, **h** 5 μm).

The morphology of the prepared materials was investigated using SEM microscopy. As shown in Fig. 2a, MCC contained large-sized fiber bundles composed of many microfibrils. These fiber clusters had an average length between 100–250 μm with an average width of 10 μm. Figure 2b,c shows the SEM images of Cu-BTC at various magnifications. These images show that Cu-BTC has an octagonal structure and a crystal size of 5 μm. This structure is consistent with the Cu-BTC synthesized in the literature^56^. As shown in Fig. 2d, the SEM image of CNC aerogels at a macro magnification (100 μm) shows the formation of an interconnected porous sheet-like cellulose network. The appearance of the sheets is related to the physical constraint of the CNC particles between the growing ice crystals. In other words, the CNCs are assembled into sheets between the growing ice crystals, forming a hierarchical macro-porous aerogel with a pore size of 10–100 μm. The CNC aerogel shows smooth pore walls at a magnification greater than 5 μm (Fig. 2e). By comparing the SEM images of the CNC aerogel (Fig. 2e), Cu-BTC (Fig. 2c), and Cu-BTC/CNC aerogel composite (Fig. 2f), a significant morphological change was observed, which might be due to the chemical cross-linking of aerogels by MBA. The MBA linkers with free electron pairs on nitrogen atoms change the morphology of Cu-BTC by coordinating open copper sites in Cu-BTC molecules and provided free space for CNC sheets to exfoliate. As seen in Fig. 2g, NFC aerogel shows a similar hierarchical porous structure to CNC aerogel structures. However, the macropores in NFC aerogels are larger than those in CNC aerogels. On the other hand, at a magnification greater (500 nm), as shown in Fig. 2h, the pore wall of NFC aerogel is not smooth but represents a lattice structure of nanofibers with macro-sized pores. That might because of the adhesion of NFC fibers is higher compared to CNC crystals. With a large number of hydroxyl groups in NFC fibers, an intermolecular hydrogen-bond network forms. Thus, NFC aerogel shrank, and consequently, the size of macropores increased. But, CNC particles, due to hemisulfate groups, have a negative charge. In Cu-BTC/NFC, the morphology of Cu-BTC did not change. As shown in Fig. 2i, several Cu-BTC particles appeared. Of course, because of the low loading of Cu-BTC 33 wt.%, Cu-BTC particles are buried inside the aerogel matrix and are not visible on its surface.

Although Cu-BTC has many applications, in this work, we have tested the performance of Cu-BTC/NFC aerogel composite in the field of water purification to ensure that Cu-BTC still retains their performance despite being trapped within the cellulose tissue. That is a promising field of application for this type of material because nanocellulose aerogels do not dissolve in water, and this aerogel can absorb more than 100 times its weight from water.

**Figure 3:**
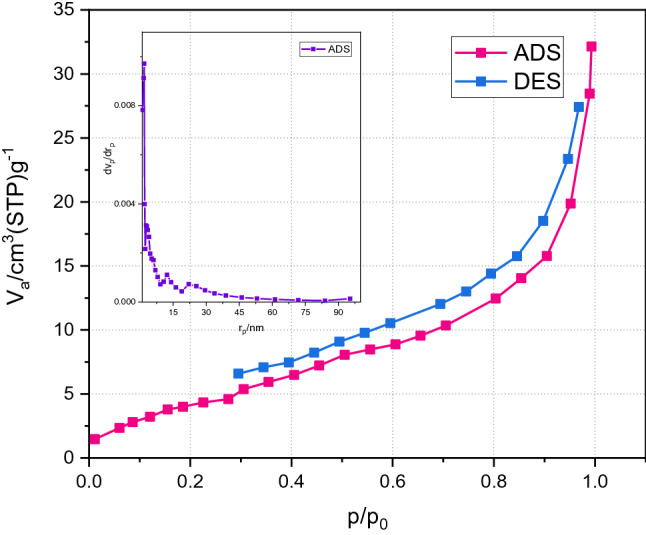
Adsorption–desorption isotherm of Cu-BTC/NFC aerogel.

To clarify the surface behavior of Cu-BTC/NFC aerogel**,** BET analysis of the compound was performed using N_2_ sorption. The N_2_ adsorption–desorption isotherm of the synthesized aerogel is shown in Fig. 3. The observed hysteresis is correlated to the typical H3 isothermal curve of mesopores (type III). The calculated specific surface area of the compound is 18.283 m^2^ g^−1^. The sharp increment in the slope of adsorption isotherm at relative low pressure (P/P0 < 0.1) indicates the presence of microspores (< 2 nm) in the material. The steady increase in the adsorption at higher relative pressures shows the monolayer/multilayer adsorption of the nitrogen molecule in the mesopores (2–50 nm), while the adsorption at the higher relative pressures (P/P0 ~ 1) indicates the total pore volume from both the micro- and mesopores. The pore size distribution by Barrett–Joyner–Halenda (BJH) analysis shown in the inset of Fig. 3, indicated that the pore diameter of Cu-BTC/NFC aerogel was in the range of mesoporous with a pore volume of 0.046 cm^3^ g^−1^.

**Figure 4:**
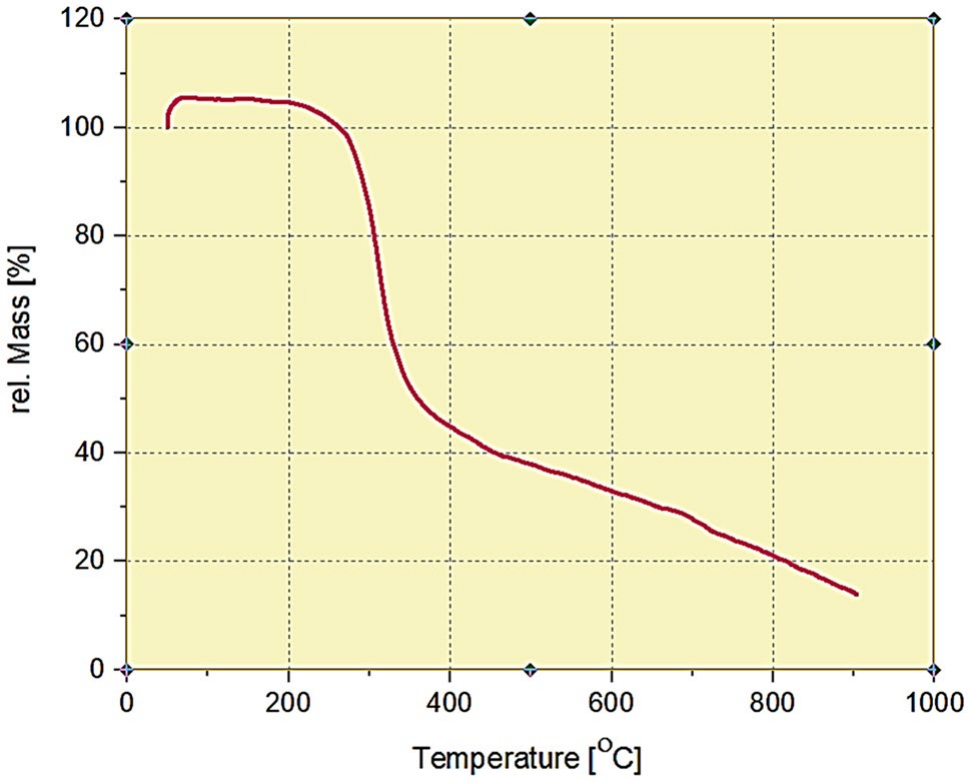
Thermal gravimetric analysis (TGA) of the Cu-BTC/NFC aerogel.

Thermal behaviors of the Cu-BTC/NFC aerogel was analyzed using TGA and the curve is plotted as a function of temperature shown in Fig. 4. The small weight losses below 150 °C for the Cu-BTC/NFC aerogel has been observed due to the evaporation of adsorbed water and the other solvents coordinated with Cu (II). The severe quality reduction stage at around 270–360 was correlated to depolymerization and decomposition of glucose units in cellulose, as previously reported^57^. Moreover, a part of the sharp weight loss of 60% from 280 to 410 °C was related to the decomposition of the trimesic acid groups of Cu-BTC, which proves the structural crash of the sample and has been reported in literature^58^.

Among the many aqueous pollutants, we chose Congo Red dye to demonstrate the ability of Cu-BTC presented in the Cu-BTC/NFC aerogel composite to adsorption. The Congo Red adsorption performance of our composite was determined by UV–Vis spectrophotometer at the wavelength of 497 nm.

**Figure 5:**
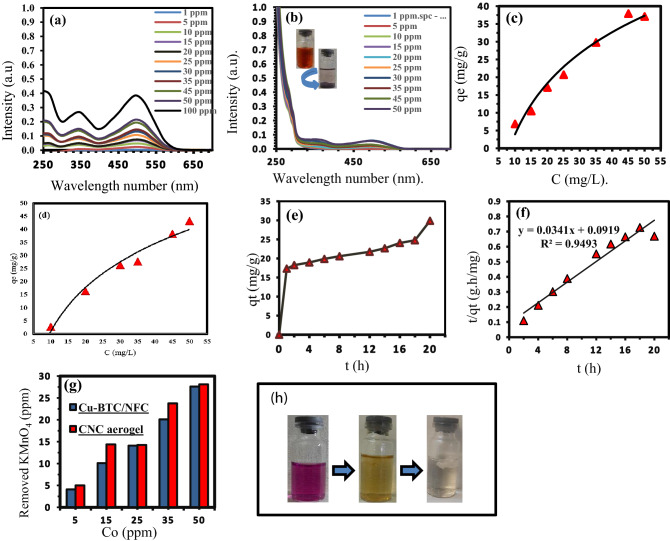
(**a**) UV–Vis spectra of aqueous solutions of Congo Red before adsorption, (**b**) after exposing to Cu-BTC/NFC. The exposing time was 18 h, (**c**) adsorption isotherm of Cu-BTC/NFC aerogel composite for various concentrations of Congo Red, (**d**) adsorption isotherm of Cu-BTC/CNC aerogel composite for various concentrations of Congo Red. (**e**) The time dependent adsorption, (**f**) correlation curve was drawn using the kinetic parameters calculated from the pseudo-second-order model, (**g**) adsorption isotherm of CNC aerogel and Cu-BTC/NFC aerogel composite for various concentrations of KMnO_4_. (**h**) The color change process from purple to colorless during the reduction of KMnO_4_.

After placing a small piece of Cu-BTC/NFC aerogel composite in the Congo Red solution, the color of the solution gradually faded to colorless, and its UV–Vis absorption maximum at 497 nm reduced significantly, as shown in Fig. 5a,b. But the color of the aerogel piece changed from sky blue to red. That means Cu-BTC/NFC aerogel composite adsorbed Congo Red molecules.

To investigate the adsorption kinetics, the adsorption capacity at different times (qt mg/g) of this composite has been obtained for Congo Red solvents with the same initial concentration. The time-dependence curve of the UV–Vis adsorption at 497 nm is fitted to the pseudo-second-order kinetic model with kinetic parameters (adsorption rate k_2_ = 0.013 g/mg h, adsorption capacity qe = 29.33 mg/g, and correlation coefficient R^2^ = 0.9493) (Fig. 5e,f).

Due to the large size of Congo Red molecules 21 Å (more than the diameter of Cu-BTC pores 9 Å), they cannot enter the Cu-BTC pores but, they were adsorbed on its surface.

To obtain the maximum Congo Red adsorption capacity by our composite, we investigate the adsorption isotherm at different initial concentrations at a constant temperature (25 °C). As shown in Fig. 5c, the equilibrium adsorption data fitted well with a Langmuir model with a maximum adsorption capacity of up to 39 mg/g. We evaluated the affinity of this aerogel for Congo Red according to the equation of K_d_ = qe/ce, and we found its distribution coefficient that K_d_ = 3546 mL/g at equilibrium concentrations ce of 11 mg/L. The results show that Cu-BTC particles in the cellulose aerogel matrix retain their function. Moreover, the adsorption isotherm of Cu-BTC/CNC aerogel was shown in Fig. 5d with a maximum adsorption capacity of 43 mg/g.

CNC aerogel did not show any adsorption for Congo Red dye. We believe since CNC aerogel shows a negative charge in aqua solution and Congo Red is an anionic dye, so due to electrostatic repulsion, CNC aerogel cannot adsorb CR molecules.

To investigate the removal of heavy metal ions, we specifically examined Cu-BTC/NFC aerogels composite and pure CNC aerogel for the elimination of potassium permanganate (KMnO_4_) shown in Fig. 5g. Although KMnO_4_ in low amounts is not toxic, and it is used excessively in water purification, however, new Canadian health research has shown that drinking water with large amounts of it can be a health hazard. It can also change the color and give an unpleasant taste of drinking water. It can also stain laundry. Therefore, it is necessary to remove permanganate ion from drinking water.

When a small piece of CNC aerogel dipped in a certain amount of aqueous solution containing potassium permanganate, the color of the solution immediately changed from purple to yellow and then to colorless with the appearance of a brown precipitate. The color of the aerogel also turned brown. After removing the piece of aerogel and filtering the solution, ICP results show that, for example, in a solution of KMnO_4_ with an initial concentration of 50 ppm, the amount of permanganate ions removed (as MnO_2_ precipitate) is 28 ppm, and there is still 22 ppm in the solution. Since the final solution is colorless, the manganese ions it contains are Mn^2+^. Accordingly, we believe that the nanocellulose aerogel substrate can reduce permanganate ions through its methylol-reducing groups.

In other words, the permanganate ions were reduced to manganese dioxide (brown precipitate) and then to manganese ions (Mn^2+^). In contrast, methylol groups in nanocellulose aerogels were oxidized to aldehyde groups and then to carboxylates.

Therefore, we conclude from this experiment that CNC aerogels did not act as an adsorbent in this case, but as standing monolith solid reductant, which could perform a reducing function without creating any byproducts in solution. Therefore, it removes KMnO_4_ by converting it to MnO_2_ precipitate.

Cu-BTC/NFC aerogel composite showed similar results but is slower compared to pure CNC. That because pure CNC aerogel was prepared under acidic conditions, and thus, it has hemi-ester sulfate groups, which act as catalysts for this ox/red reaction.

## Conclusions

In brief, we successfully synthesized pure nanocellulose aerogels and also Cu-BTC/nanocellulose aerogel composites. The nanocellulose aerogel acts as a standing mold for Cu-BTC powder, and Cu-BTC trapped in the nanocellulose aerogel maintains its performance. As a result, the as prepared composite showed good absorption for Congo Red. Nevertheless, pure CNC aerogel did not show any adsorption for Congo Red because of its anionic properties in an aqueous solution. On the other hand, both Cu-BTC/NFC aerogel composite and pure CNC aerogel act as standing monolith solid reductants. We believe that a standing monolith solid reductant will attract a great deal of research in the future. Because it can perform its application without creating any hazardous byproducts in the solution and accordingly, it avoids the need for the separation of byproducts. On the other hand, it is easy to separate it from the reaction medium. Most importantly, depending on the synthesis conditions, this type of compound can play the role of the reductant and the catalyst simultaneously, like in the case of the CNC aerogel.

## Experimental section

### Materials

Peanut shells were purchased from a grocery store and used as the raw materials. Sodium hypochlorite solution (6–14%), copper nitrate trihydrate (Cu(NO_3_)_2_·3H_2_O), sulfuric acid (95–98 wt%), 1,3,5-benzene tricarboxylic acid (BTC), *N*,*N'-*methylene bisacrylamide (MBA), and other reagents (NaOH, HNO_3_, CHCl_3_, EtOH, *t*-butyl alcohol and Congo Red) were all purchased from Merck. Deionized water (resistivity 18.2 MΩ/cm) was used in all cases. All the chemicals in this study were used as received without any further purification.

FTIR measurements were conducted on Nicolet 6700 Fourier transform infrared spectrometer. Scanning electron microscopy (SEM) measurements were conducted on Hitachi S-4800 instruments operated at 2 kV for gold-sputtered samples. XRD patterns were recorded on a Bruker D8 ADVANCE X-ray diffractometer with a Cu Ka radiation (λ = 1.5418 Å). The powder was leveled on sample holders and scanned with a 2θ angle from 5° to 50° with a step speed of 5°/min. The concentration of the contaminated water was determined using a DU 800 UV–Vis spectrophotometer. The amounts of Mn^2+^ and Mn^7+^ were determined by Inductively Coupled Plasma (ICP) analysis on sequential plasma spectrometer, Shimadzu (ICPS-7000).

### Isolation of microcrystalline cellulose MCC

MCC was extracted according to the literature with some modifications^25^. In brief, the peanut shells were washed with running water and oven-dried over 2 days at 60 °C to remove moisture. And then, we ground the dried shells into powder form using a mill. 25 g of the powder was treated with NaOH (750 mL, 0.5 M) for 3 h at 95 °C with continuous stirring. The dark slurry obtained was filtered and washed several times with distilled water and then dried. The dried powder refluxed for 7 h with a mixture containing 20% (V/V) OF (HNO_3_ in EtOH), then the color changed from brown to yellow. The mixture was then filtered and washed with cold distilled water till the solution becomes neutral. Sodium hypochlorite was used to get-off the colored residue into white cellulose. It was then oven-dried overnight at 50 °C to constant weight.

### Preparation of crystalline nanocellulose CNC by acid hydrolysis

CNCs were prepared according to previous methods^25^. Briefly, 2 g of microcrystalline cellulose MCC was mixed with 18 mL of H_2_SO_4_ aq. (64% wt.), and continuously stirred at 50 °C for 2 h. The mixture was diluted with cold deionized water to stop the hydrolysis, and the cloudy suspension was centrifuged at 9000 rpm several times to remove acid and soluble hydrolysates. The resultant CNCs washed up until the pH of the solution became 2.3.

### Preparation of nanofibrillated cellulose NFC

About 1 g of MCC was dispersed with 35 mL of distilled water to form a dispersion. The resulting dispersed was treated with high-intensity ultrasonic (HIUS) for 1 h to transform into a transparent gel of cellulose nanofibers (NFC). The resulting hydrogel was kept in the refrigerator at 4 °C for subsequent work.

### Preparation of CNC aerogel

Nanocrystalline cellulose aerogel was chemically cross-linked mostly by *N,N'-*methylenebisacrylamide (MBA). CNC was first dispersed in 30 mL of distilled water to form a suspension, and then 1 g of MBA was added to the mixture with continuous stirring for 1 h. After that, it was treated with ultrasonic at 25 °C for one hour to transform into a gel. The resulting gel was aged for 15 h to increase its strength and provide the opportunity to form chemical bonds between the functional groups of nanocrystalline cellulose (sulfate hemi-ester) and organic linker. Finally, the gel froze at − 20 °C for 2 days and freeze-dried to obtain the aerogel.

### Preparation of NFC aerogel

The NFC aerogel was cross-linked physically by hydrogen bonding and entanglements. It was washed three times with EtOH, solvent-exchanged in *t*-BuOH, and freeze-dried to get aerogel. During the solvent-exchanging process to avoid shrinking NFC hydrogel, it immersed in the mixed solution of EtOH and *t-*BuOH, which *t*-BuOH content increased step by step. Subsequently, the gel was frozen at − 20 °C for 2 days before freeze-drying.

### Synthesis of Cu-BTC

Cu-BTC was synthesized by a solvothermal method according to the synthesis procedure reported elsewhere with some modifications^34^. First, Cu(NO_3_)_2_·3H_2_O (1.94 g) dissolved in deionized water (24 mL). BTC (benzene 1,3,5-tricarboxylic acid, 0.84 g) dissolved in EtOH (24 mL). The two above solutions were mixed and stirred at room temperature until forming a suspension. Second, the mixture was transmitted into a Teflon-lined stainless steel reactor and placed in an oven 110 °C for 28 h. Then, the reactor was allowed to cool naturally to room temperature. A dark blue solid was obtained as the major product, which was then washed separately with EtOH, DMF, and CHCl_3_. Finally, the solid was collected and heated at 100 °C for 18 h in a vacuum oven to obtain a fine powder of Cu-BTC.

### Fabrication of Cu-BTC/CNC aerogel composite

Cu-BTC/CNC aerogel was prepared by the direct-mixing method. Cu-BTC powder was mixed with CNCs suspension and stirred forcefully for 30 min to obtain a homogeneous dispersion. 1 g MBA was added to the dispersal with continuous stirring for 1 h. After that, it was treated with ultrasonic at 25 °C for 1 h to transform into a gel. This gel was aged for 15 h to increase its strength providing the opportunity to form chemical bonds between the functional groups of nanocrystalline cellulose (sulfate hemi-ester) and organic linker. Finally, the gel was frozen at − 20 °C for 2 days and freeze-dried to obtain the aerogel.

### Fabrication of Cu-BTC/NFC aerogel composite

Cu-BTC nanoparticles were mixed with NFC gel and stirred forcefully for 1 h to obtain a homogenous mixture. After that, the resulting mixture was washed with ethanol three times, solvent-exchanged in *t-*BuOH, and freeze-dried. During the solvent-exchanging process to avoid shrinking Cu-BTC/NFC hydrogel, it was immersed in the mixed solution of EtOH and *t*-BuOH, which *t-*BuOH content increased step by step. Subsequently, the gel was frozen at − 20 °C for 2 days before freeze-drying.

### Adsorption evaluation of Cu-BTC/NFC aerogel composite and CNC aerogel

The adsorption isotherm experiments were navigated by putting 0.01 g adsorbent in 10 mL of Congo Red dye solution of various initial concentrations (1, 5, 10, 15, 20, 25, 30, 35, 45, and 50 ppm).

Adsorption kinetics were carried out by soaking the adsorbent in 10 mL of an aqueous solution containing 30 mg L^−1^ Congo Red for a predetermined time. The solution was then analyzed by UV–Vis spectroscopy to determine the concentration based on a calibration curve prepared from a solution with known Congo Red concentration. The adsorption capacity at equilibrium qe (mg/g) and at time t qt (mg/g) were calculated using the following equations, respectively:

$$ {\text{q}}_{{\text{e}}} = \frac{{\left( {{\text{C}}_{0} - {\text{C}}_{{\text{e}}} } \right) V}}{M} \quad {\text{and}}\quad {\text{q}}_{{\text{t}}} = \frac{{\left( {{\text{C}}_{0} - {\text{C}}_{{\text{e}}} } \right) V}}{{\text{M}}} $$

where C_0_, C_e_ and C_t_ are the liquid phase concentrations of the Congo Red dye at initial, equilibrium and at any time t respectively (mg/g), V is the volume of the solution (L) and M is the mass pf the adsorbent (g).

The time-dependence of the adsorption was fitted with the pseudo-second-order kinetic model which was:

$$ \frac{t}{q_{t}} = \frac{1}{{K_{2}{q_{e}}^{2}}} + \frac{1}{q_{e}}t $$

where q_t_ and q_e_ were the adsorption capacities at time t and at equilibrium respectively, and the K_2_ is the rate constant of the pseudo-second-order model.

The adsorption of KMnO_4_ was studied by putting a piece of aerogel (pure CNC aerogel or Cu-BTC/NFC aerogel composite) in a solution with different initial concentrations. When the solution became colorless, it was filtered, and ICP-OES analysis was performed.
